# Gene Expression Detects the Factors Influencing the Reproductive Success and the Survival Rates of *Paracentrotus lividus* Offspring

**DOI:** 10.3390/ijms232112790

**Published:** 2022-10-24

**Authors:** Serena Federico, Francesca Glaviano, Roberta Esposito, Bruno Pinto, Maissa Gharbi, Anna Di Cosmo, Maria Costantini, Valerio Zupo

**Affiliations:** 1 Stazione Zoologica Anton Dohrn, Department of Ecosustainable Marine Biotechnology, Via Ammiraglio Ferdinando Acton n. 55, 80133 Napoli, Italy; 2 Stazione Zoologica Anton Dohrn, Department of Ecosustainable Marine Biotechnology, Ischia Marine Centre, 80077 Napoli, Italy; 3 Department of Biology, University of Naples Federico II, Via Cinthia, 80126 Naples, Italy; 4 General Fisheries Commission for the Mediterranean (GFCM) Palazzo Blumenstihl Via Vittoria Colonna, 1, 00193 Rome, Italy

**Keywords:** aquaculture, carryover effects, skeletogenesis, stress response

## Abstract

The increase in the demand for *Paracentrotus lividus* roe, a food delicacy, causes increased pressure on its wild stocks. In this scenario, aquaculture facilities will mitigate the effects of anthropogenic pressures on the wild stocks of *P. lividus*. Consequently, experimental studies should be conducted to enhance techniques to improve efficient aquaculture practices for these animals. Here, we for the first time performed molecular investigations on cultured sea urchins. We aimed at understanding if maternal influences may significantly impact the life of future offspring, and how the culture conditions may impact the development and growth of cultured specimens. Our findings demonstrate that the outcomes of in vitro fertilization of *P. lividus* are influenced by maternal influences, but these effects are largely determined by culture conditions. In fact, twenty-three genes involved in the response to stress and skeletogenesis, whose expressions were measured by *Real Time qPCR*, were differently expressed in sea urchins cultured in two experimental conditions, and the results were largely modified in offspring deriving from two groups of females. The findings herein reported will be critical to develop protocols for the larval culture of the most common sea urchin, both for research and industrial production purposes for mass production.

## 1. Introduction

### 1.1. Sea Urchins and Aquaculture

Sea urchins are a resource for the scientific research, besides their key role in the ecology of Mediterranean shallow ecosystems and their fishery importance. In fact, the Mediterranean *Paracentrotus lividus* has both an ecological and an economic importance, and an emerging demand for fresh animals characterizes the fish markets, because their gonads (also known as “roe”) are considered a food delicacy worldwide and a substantial source of revenue. The roe consists not only of immature and mature gametes but also of germinal and connective tissues. Consequently, the number of gametes is not the only factor related to gonadal size, because the growth of nutritive phagocytes and accessory tissues also influence the size of gonads. This is important because it is possible to enhance the gonadal growth of farmed individuals by favoring the accumulation of nutrients independently from their actual reproductive power [1]. Aquaculture of echinoderms (sea urchins and sea cucumbers) is defined as “echinoculture”, but this term often refers specifically to sea urchin culture in the laboratory. Wild populations of *P. lividus* are presently facing changing environmental conditions and overexploitation of their stocks. This issue urgently needs action to ensure correct management of the resource. The implementation of eco-friendly and efficient aquaculture systems could help in overcoming the overexploitation of marine resources, including sea urchins. In addition, sea urchins play a pivotal role in scientific research [2] because they are widely used for ecotoxicological, physiological and embryological studies due to their easy handling in the laboratory and to the transparency of their embryos, ensuring that the first developmental stages can be easily monitored [3,4,5]. Sea urchin larvae are also employed as live feed for other cultured organisms, such as fishes and filter feeders (e.g., shellfish larvae). Consequently, they represent a valuable resource for the culture of other economically-relevant species [6].

### 1.2. Factors Influencing Larval Development

Several factors, such as diet composition, larval density and quality of the culture environment, may deeply impact the reproductive success of this species [7,8,9,10]. Variable feed abundance, feeding frequencies and species of live microalgae used for larval diets can severely influence larval growth, survival and metamorphic success [8,9,11,12] according to the cultured species of sea urchin. In addition, the nutritional quality of microalgae can vary depending on culture factors influencing their cell size, digestibility and biochemical composition [13]. For example, *Chaetoceros* spp. and *Tisochrysis lutea* contain relatively high amounts of long-chain PUFAs, but neither are rich in both EPA and DHA [14], and in their research were found to decreasein the absence of *Rhodomonas lens*. Castilla-Gavillán et al. [10] demonstrated that *P. lividus* larvae fed on *Rhodomonas* sp. contained higher total lipid content than those fed on other microalgae. Similar dynamics could have positive implications for the development of larval echinoderms. Previous studies [15] indicated that *Dunaliella* sp. may be sufficient to sustain the complete development of *P. lividus* up to metamorphosis, although small additions of other feeds (e.g., *R. lens*) may represent a useful improvement towards complete success of the larviculture. In contrast, some microalgae may have deleterious effects on the growth of post-larvae [16].

However, maternal and zygotic factors also influence and control the early development of sea urchins. Maternal factors include messenger RNA and proteins expressed during oogenesis. mRNA is also necessary before activation of the embryonic genome for early development, as they are involved in the regulation of metabolism, cell cycle and development [17]. When genome activation occurs in embryos, zygotic factors begin to be expressed. The first known developmental genes are expressed at the reaching of 16-cell stage; before this stage any developmental process is completely driven by maternal factors [18,19]. Besides their genetic contribution, mother sea urchins control the phenotypic development of their offspring in response to environmental conditions [20]. These maternal influences, which result in the combined effect of maternal phenotype and genotype [21], can have significant effects on the fitness and performance of the offspring [22,23,24]. In addition, they can influence the sea urchin population ecology [21,25]. However, factors experienced by sea urchin females during early development can also affect the phenotypes and fitness of their offspring.

In addition, the size of culture vessels and the density of larvae may represent key factors in determining rates of survival of larvae up to metamorphosis. An experiment conducted in small-scale 1.3-L culture vessels revealed an inverse relationship between larval density of a sea urchin (*Diadema antillarum*) and growth [26]. Previous authors obtained variable rates of survival by approaching different larval-rearing systems [27]. Different studies were characterized by variable feeding protocols, fertilization techniques and rearing devices [28]. In particular, previous research has indicated culture experiences within tanks of different volumes, besides the influence of additional rearing devices (e.g., aeration systems, palettes, procedures, etc.). Hence, we aimed to investigate if tanks of the same shape but different sizes might influence the survival of larvae. In fact, keeping constant the larval density and the concentration of microalgae feed, tanks of different volumes are characterized by variable hydrodynamic conditions. For example, larger tanks need faster bubbling to ensure sufficient gas exchanges, when compared to smaller tanks, but aeration may interfere with the limited swimming capabilities of planktonic larvae, pushing them into some sectors on the bottom. However, an appropriate determination of the most favorable stocking density within a production-oriented recirculating aquaculture system (RAS) has not been performed. Consequently, the size of culture vessels, the quality of larval nutrition and the stocking density remain extremely relevant for the larviculture of sea urchins, especially when considering the feasibility of production for fisheries, restoration and scientific research. For these reasons, we tested the effect of the tank size in order to detect any influence on survival rates during the early development of these sea urchins.

We had two distinct aims in our manuscript: (i) we aimed at understanding if maternal influences may significantly impact the life of future offspring; (ii) we aimed at detecting how and if slight variations in the culture conditions may impact the development and growth of cultured specimens. In addition, we aimed at evaluating the best production sizes, in terms of tank volumes, to ensure correct growth and higher survival rates of *P. lividus* embryos. On the whole, we aimed at understanding how genic resources interact with environmental stressors to ensure maximum reproductive performance in a marine invertebrate. Furthermore, and for the first time, we tested the effect of these production units by performing molecular analyses (*Real-Time qPCR*) to check the expression levels of several genes involved in stress responses and skeletogenesis. In particular, we followed the variation of expression levels of twenty-three genes, which were first isolated in sea urchin embryos in response to different stressors [3,5,29,30,31,32,33]. In this study, gene expression levels are not responses related to a stimulus but must be intended as higher or lower levels of gene expression.

## 2. Results

### 2.1. Survival Rates

**Figure 1:**
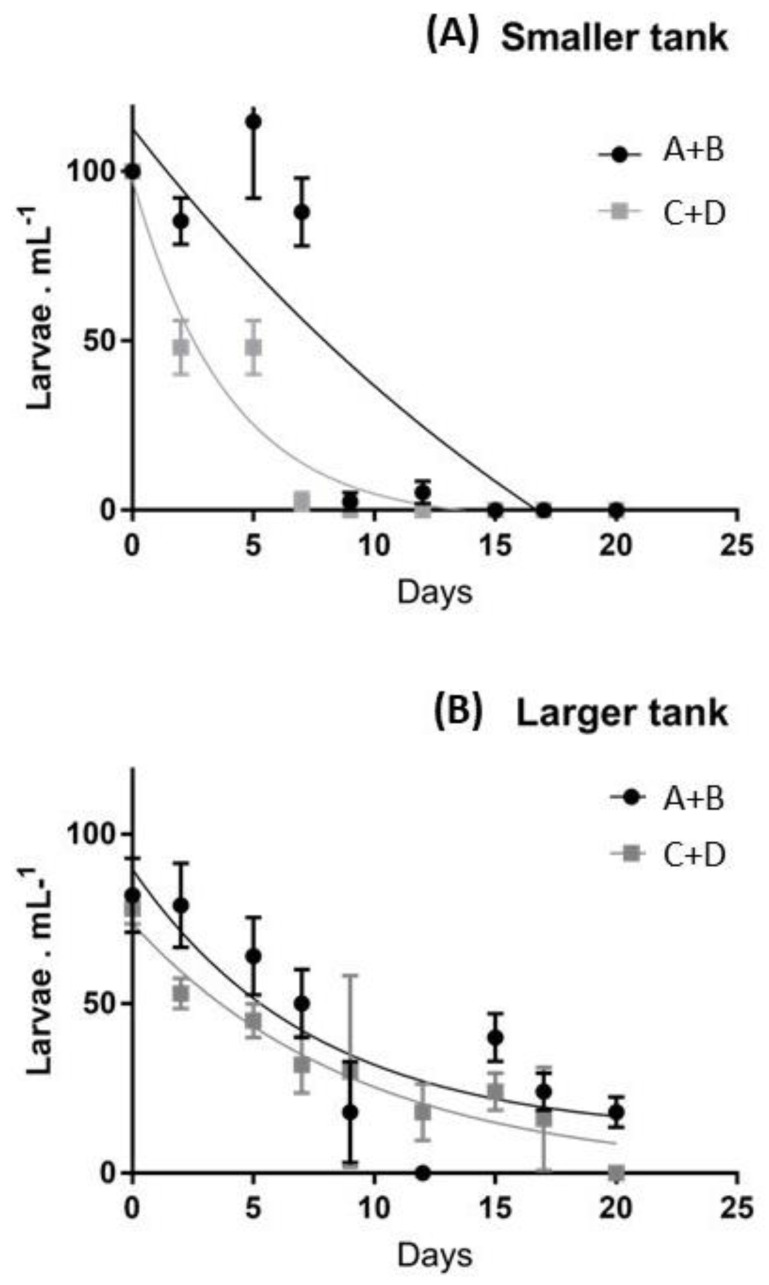
Average survival rates of larvae obtained from the two pools of individuals (A+B vs. C+D) in smaller (**A**) and larger (**B**) tanks.

Even if the survival rates of larvae deriving from females A-B in the first three weeks of growth were not significantly different from those deriving from females C–D, when analyzed by means of paired *t*-tests (Table 1):

The performances of the two sets of larvae exhibited different patterns of development (Figure 1).

In particular, pool A-B exhibited higher survival rates in the smaller tanks (Figure 1A), completing the settlement after 17 days. In contrast, pool C-D exhibited lower survival rates during the first two weeks and the number of swimming larvae was strongly reduced after two weeks, due to higher mortality. In fact, in the last two weeks, larvae were absent in both groups of females considered. These differences were less evident in larger tanks (Figure 1B), where the number of larvae continuously decreased in the first 20 days, exhibiting a low number of settled individuals in both A-B and C-D groups (Figure 1B). The larval stage progression was initially similar in the two pools and the embryonic stages developed synchronously through the pluteus stage. However, from the onset of feeding through the larval stages, the development became increasingly less synchronous between pools A-B vs. C-D, and even among tanks of different sizes. After the first week, larvae from the pool A-B exhibited faster development, showed dark-green guts and after 10–12 days reached a six-arm stage, while larvae from the pool C-D exhibited slower development, about half of them arrested their development at a four-arm stage, and were characterized by partially-empty guts. In particular, larvae cultured in smaller tanks reached the advanced rudiment stage after about two weeks, while larvae in larger tanks showed lower mobility, sank to the bottom more often and their guts exhibited a pale color.

**Figure 2:**
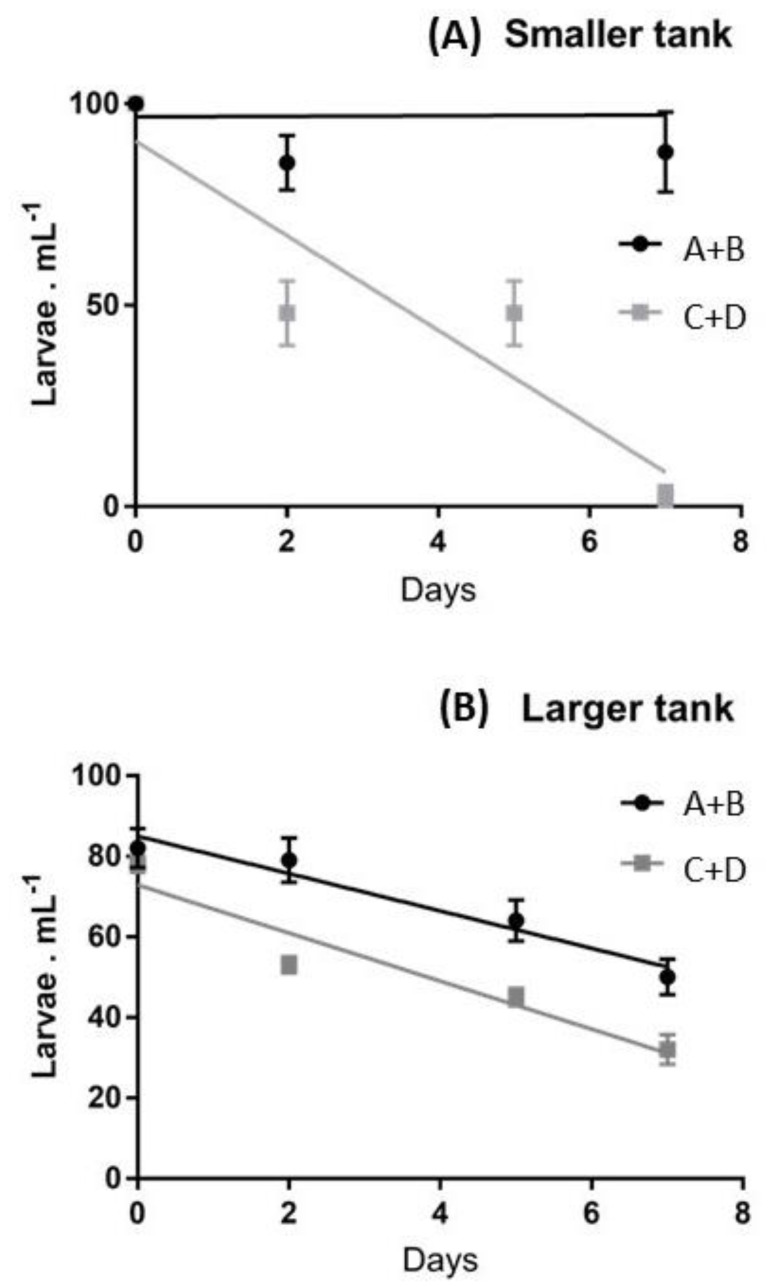
Average larval survival rates evaluated during the first week in (**A**) smaller and (**B**) largertanks, referring to the recruits of females A+B vs. those of females C+D. The linear regressions are superimposed.

To evaluate the effect of maternal influences and the effect of tank size on the survival of larvae (removing the influence of settlers on the density of swimming larvae) only the first week of culture was analyzed and the slopes of linear regressions were compared (Figure 2).

This representation permits the evaluation that in smaller tanks, during the first week, an 100% survival was exhibited by larvae deriving from females A and B, whilst, in the same period, females C and D produced a decline of larvae, down to 2.6% of the initial pool. When slopes were compared, the differences in the survival patterns of the two larval pools were significant at *p* < 0.05. In larger tanks a continuous decrease of the swimming larvae was observed during the first week for both larval pools (Figure 2B) and the differences in the survival patterns of the two larval pools were not significant (*p* > 0.05).

### 2.2. Molecular Analyses of Embryos Deriving from Various Females

The variation of expression levels of twenty-three genes, involved in the stress response and skeletogenesis and previously analyzed [3,5,34,35,36,37,38] (see Table S3) was followed by *Real Time qPCR* (see Table S4).

#### 2.2.1. Genes Involved in Stress Response

Concerning the 18 genes analyzed, the results show that the plutei deriving from female A did not have variations in expression for any of the genes analyzed. The same results were found for the plutei deriving from female B, with the only exception being *CASP8* gene, which decreased its expression level. In the case of plutei deriving from female C, fifteen genes decreased their expression levels: *ARF1* (−3.5), *CASP8* (−5.5), *caspase 3/7* (−3.5), *ERCC3* (−3.9), *GRHPR* (−2.4), *hsp60* (−2.4), *hsp70* (−6.4), *HIF1A* (−2.0), *PARP-1* (−3.0), *p53* (−4.8) and *14-3-3 ε* (−5.5). Only *cytb* gene showed an increase (2.5) of its expression level. Moreover, in the plutei deriving from female D, nine genes decreased their expression levels (*CASP8*, −2.7; *ERCC3*, −3.2; *hsp60*, −2.8; *hsp70*, −2.9; *p38MAPK*, −2.3; *SDH*, −2.7; *p53*, −4.4; and *14-3-3 ε*, −3.4).

#### 2.2.2. Skeletogenic Genes

None of the five genes analyzed was switched on in the plutei deriving from females A and B. Three genes were down-regulated in the plutei deriving from female C (*SM30*, −3.9; *SM50*, −4.0; *uni*, 4.0), whereas plutei deriving from female D showed a down-regulation of *SM50* (−7.3) and an up-regulation of *univin* (4.0).

### 2.3. Network Analysis

Interactomic analysis showed that eighteen genes of the twenty-three analyzed from *Real Time qPCR* are connected by functional point of view: ADP-ribosylation factor 1 (*ARF1*), caspase-8 (*CASP8*), Sp-Cspe3/7L (*CASPASE 3/7*), cytochrome b (*CYTB*), DNA-methyltransferase 1 (*MTase*), ERCC excision repair 3 (*ERCC3*), glutamine synthetase (*GS*), glyoxylate reductase/hydroxypyruvate reductase (*GRHPR*), heat shock protein 56 (*HSP56*), heat shock protein 60 (*HSP60*), heat shock protein 70 (*HSP70*), hypoxia inducible factor 1-alpha (*HIF1A*), *nuclear factor kappa light-chain-enhancer of activated B cells* (*NF-kB*), Poly(ADP-ribose) polymerase 1 (*PARP-1*), p38 mitogen-activated protein kinase (*p38 MAPK*), succinate dehydrogenase (*SDH*), tumor protein p53 (*p53*). Only the skeletogenic genes 14-3-3 epsilon protein (*14-3-3 ε*), bone morphogenetic protein 5-7 (*BMP5-7*), and nectin (*Nec*) weren’t included in network analysis because they have no orthologous genes in humans. Moreover, GRHPR is not linked to any gene under analysis.

**Figure 3:**
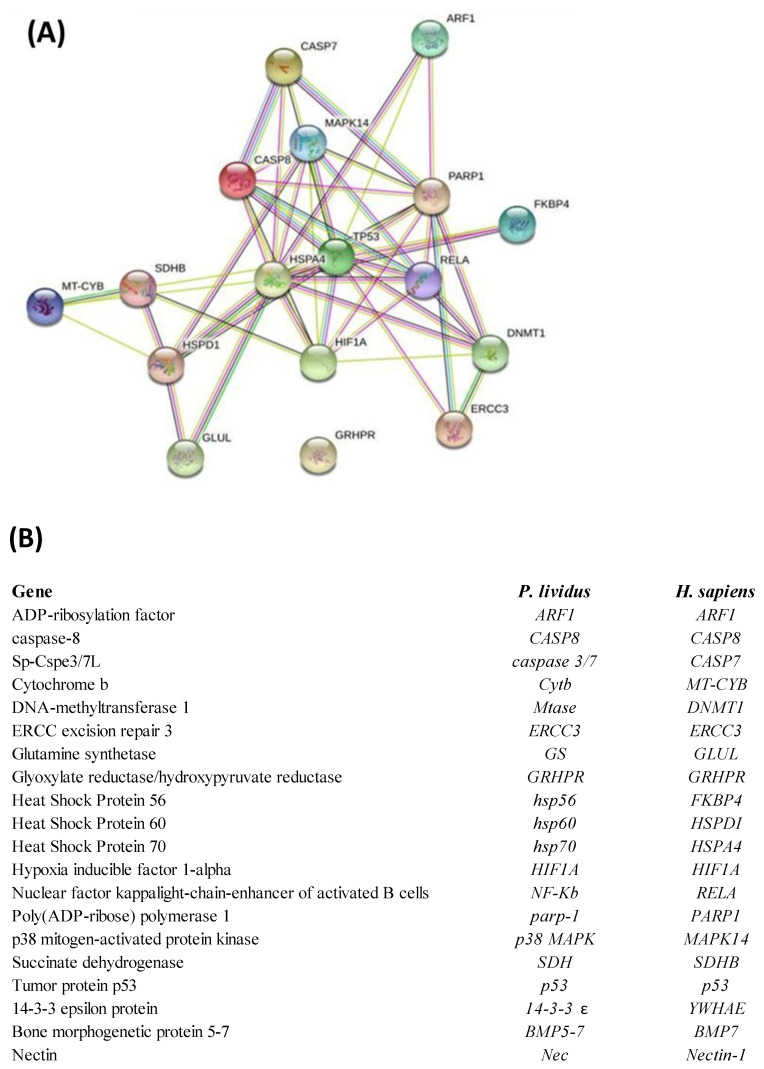
(**A**) Interactomic analysis by STRING (https://string–db.org/; accessed on 30 April 2022). The network graphically displays the relationship between genes. The biological relationships between genes are indicated by different colors. Known interactions: reported by database = light blue and determined experimentally = pink. Expected interactions: gene proximity = green; gene fusion = red; and genes with similar pattern = light blue. (**B**) *Homo sapiens* gene names and the corresponding *P. lividus* orthologous genes. The most significant relations among genes (confidence score cut-off = 900) displaying experimental evidence are highlighted.

As shown in the network reported in Figure 3, the genes are correlated as follows: *ARF1* with *hsp70*, *p53* and *PARP1*; *Caspase 3/7* with *CASP8*, *hsp70*, *p38 mapk*, *PARP1*; *CASP8* with *Caspase 3/7*, *p38 mapk*, *hsp70*, *p53*, *NF-kB*, *PARP1*; *p38 mapk* with *CASP8*, *Caspase 3/7*, *hsp60*, *hsp70*, *p53* and *PARP1*; *Hsp70* with *CASP8*, *p53*, *NF-kB*, *MTase*, *HIF1A*, *hsp60*, *SDH*; *p53* with *p38 mapk*, *CASP8*, *hsp70*, *HIF1A*, *ERCC3, NF-kB, MTase, PARP1, hsp56; PARP1* with *ARF1, Caspase 3/7, p38 mapk, CASP8, p53, HIF1A, NF-kB, ERCC3* and *MTase; Hsp56* with *p53; NF-kB* with *PARP1, p38 mapk, p53, hsp70, HIF1A, ERCC3, MTase; MTase* with *PARP1, NF-kB, p53, hsp70, ERCC3; ERCC3* with *MTase, PARP1* and *p53; HIF1A* with *NF-kB, PARP1, MTase, p53, hsp70* and *SDH; SDH* with *hsp70, HIF1A, hsp60* and *CytB; CytB* with *SDH* and *hsp60; Hsp60* with *CytB, SDH, p38 mapk, hsp70, p53* and *GS; GS* with *hsp60* and *hsp70*. The corresponding human orthologous genes are also reported.

## 3. Discussion

Investigations of other echinoderms reported that the performance of one life history stage led to (positive or negative) carryover effects on the subsequent life stages [39]. In fact, carryover effects arising within a generation can influence next generations [40,41,42]. Carryover effects can also arise across a generation, an effect known as transgenerational plasticity (TGP) [43]. Due to environmental stresses [44] experienced by one or both parents during the development of their gametes [23,41], a transgenerational response can lead to phenotypic changes in the offspring [43,45]. Various evidence indicates that long-term exposure of adult sea urchins to thermal shocks impacts the embryo development of their offspring [39,46,47]. Here we aimed at checking and eventually confirming this hypothesis regarding *Paracentrotus lividus* embryos and larvae.

During the whole experiment the main abiotic descriptors were kept stable and did not achieve significant variations among replicates and treatments. Maternal effects on the survival rates of larvae were definitely observed in smaller culture vessels. Larger vessels prompt a negative influence on the survival, which is likely to be superimposed onto the material influence itself. This evidence indicates that both the maternal effects and the size of the culture tanks are critical to determine the reproductive success of this species, but the influence of the latter overwhelms that of the former.

Evidently the size of the tank matters, because the two experimental conditions produced significantly different results in terms of survival rates of larvae. The effect might be explained by taking into account that circulation of water and oxygen stratification change drastically according to the shape and the size of the culture vessels. In particular, deeper tanks exhibit a lower circulation of water and a higher stratification of gases, because the rate of aeration (air bubbling) cannot be proportionally changed to avoid damage to the delicate larvae swimming closer to the bubbling tubes [48,49,50]. We might also consider the need for larvae to remain in the upper levels and to avoid strong contact with the bottom of the tank, since it may contain algal and bacterial films which produce mucilage that promotes clogging and aggregation of larvae (personal observation; [51]). Since larger tanks require higher levels of aeration to guarantee sufficient gas exchanges, the fluxes produced by the air stone also promote the creation of sink areas where larvae aggregate close to the bottom. In contrast, the aeration may be very weak in the smaller tanks, permitting the larvae to swim towards the surface without interference. In fact, in smaller containers most larvae were continuously visible under the surface of the water, while in larger tanks they were actively transported by the currents and could accumulate in areas with lower perturbations, close to the bottom (personal observation). Attempts to reduce this effect by decreasing the flux of the air produced lower gas exchanges which were insufficient to guarantee consistency of the abiotic factors (including the saturation of oxygen in the seawater) that represented a pre-condition to evaluate other sources of stress [49,52].

As reported, the density of larvae deriving from females C-D exhibited a decrease in smaller tanks, as opposed to larvae deriving from females A-B, which performed best. In contrast, both groups of larvae showed mortality rates higher than 60% in the first week when cultured in larger tanks, and the differences were not significant in this condition. This may lead to the conclusion that the first factor determining survival rate is represented by the environmental conditions and that, in an optimal environment, maternal influences become determinant. The overlap of the growth conditions to the maternal influence may also explain the interannual differences in larval recruitment of this species observed in the field [53].

**Figure 4:**
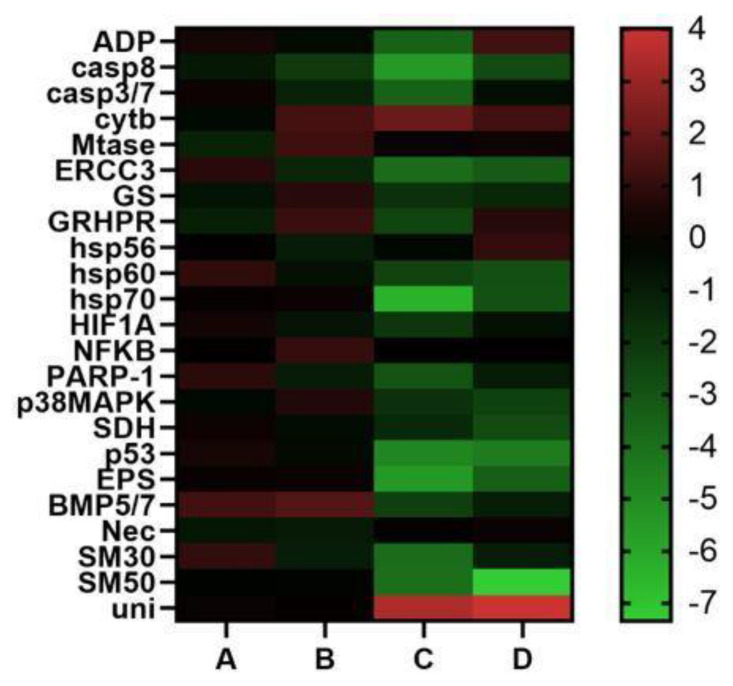
Heatmaps (Heatmapper available at www.heatmapper.ca, accessed on 28 July 2022) showing the expression profiles and hierarchical clustering of twenty-three genes analyzed by *Real Time qPCR* in *P. lividus* embryos deriving from females A, B, C and D. Color code: red = up-regulated genes with respect to the control; green = down-regulated genes.

Molecular analyses were performed to understand if there were differences in the levels of gene expression associated with offspring deriving from different *P. lividus* females (see Figure 4). Molecular data strongly supported the low success of embryonic development for the plutei deriving from females 3 and 4. In fact, plutei deriving from females A and B did not show significant variations in their gene expressions.

In contrast, plutei deriving from females C and D exhibited a remarkable switch on in almost all the genes analyzed that are involved in stress response and in skeletogenic process, indicating negative maternal influences, as showed by the results of *Real Time qPCR*. In fact, the down-regulation of genes in the plutei deriving from *P. lividus* females C and D was evident compared to those deriving from females A and B. These effects in marine organisms are not new [23], because they have been widely investigated in various sea urchin species [54,55,56], but to the best of our knowledge such relationships have never been investigated in *P. lividus* [17].

Interactomic analyses showed that stress genes were functionally correlated among themselves. *GRHPR* was the only gene that appeared to have no correlation with others. Previous investigations indicated that this gene is correlated to *HSPA4* and *GLUL* through *CAT* [38], which was also analyzed in this research.

Changes of gene expression induced by environmental stressors on somatic cells can affect the physiology of exposed individuals. As a consequence, some alterations can be propagated to subsequent generations through the germline, occurring during the gametogenesis [57,58,59]. In particular, environmental stresses experienced by one or both parents may lead to aberrant patterns in F1, which can become evident from the early developmental stages [56,60]. It is known that a network of regulatory genes, the so called “defensome” [3,30,61], regulate the mechanisms of specification and differentiation during embryonic development.

Our findings show how changes in the levels of gene expression may be used as an early indicator of stressful conditions for sea urchins, and more generally for marine invertebrates and the marine environment itself. Furthermore, these findings demonstrate that changes in gene expression can help understand the health state of adult sea urchins. The identification of key genes and the molecular pathways in which they are involved represent fundamental tools for understanding how marine organisms attempt to protect themselves against toxicants in order to avoid deleterious consequences and irreversible damage. Furthermore, changes in the response to environmental stressors may contribute to an adaptive survival advantage of organisms through beneficial gene expression [39].

The findings herein demonstrated will be useful for the culture of the most common Mediterranean sea urchin, both for research purposes and for the needs of aquaculture. In fact, despite several established methods of commercially producing echinoderms from gametes [62,63], a scalable hatchery process for *P. lividus* has not yet been established. Attempts to culture this species over the last decades have been met with varying degrees of success, and speculative factors including environmental toxins, water quality and general culture methods precluded reliable development through metamorphosis [26]. This study was intended to develop *P. lividus* larval culture protocols within a laboratory or an industrial production plant capable of scaled production for aquaculture, restoration or scientific research. The culture conditions demonstrated are adequate for the larval development of *P. lividus,* but variables of interest, i.e., maternal influences and size of the culture vessels, are demonstrated to be critical factors to reach sufficient survival rates and complete development of this species. However, further investigations aimed at improving yields are necessary, as well as to understand how larval nutrition affects settlement and post-settlement success in order to further improve commercial production viability.

## 4. Materials and Method

### 4.1. Gamete Collection and Embryo Culture

Adult sea urchins were collected by scuba divers around the island of Procida (Italy) in February 2021, at 10 m depth. Collected specimens were transported in an insulated box to the laboratory and maintained in tanks with circulating sea water for ten days until testing. Sea urchins were injected with 1 mL of 2 M KCl through the peribuccal membrane to stimulate the emission of gametes. After the production of gametes, the specimens were restored in aerated recirculated tanks and released after a few days into the same area of collection. Eggs were collected in a glass dish, washed with filtered sea water (FSW) and kept in FSW until use. Concentrated sperm was collected from males by means of a plastic pipette and kept undiluted at 4 °C until use.

Eggs were fertilized and were kept at 20 °C in a controlled temperature chamber on a 12 h–12 h light–dark cycle. Forty-eight hours post-fertilization the embryos reached the pluteus stage, and these larvae were distributed in equal concentrations (1.5 plutei mL^−1^) in triplicate tanks of two different volumes: (i) small scale, in 2 L beakers; (ii) larger scale, in 10 L tanks. Embryos deriving from individual females were paired two by two and kept separate, aiming at detecting any variation due to maternal influences and if the initial quality of the gametes influenced the larval development. In particular, the eggs deriving from four females were randomly pooled into two groups (females A-B and females C-D) prior to being fertilized with a pool of sperms deriving from five males. Four females were randomly chosen because TR-qPCR technique is useful to detect individual variations in the gene expression, avoiding the flattening of results due to variable gene expressions detected in larger populations.

Larvae were fed on *Dunaliella tertiolecta* (5000 cells ml^−1^; [15]) along with a composed algal food (SHG Snow Reef, Super High Group, Ovada, Italy; www.superhigroup.com accessed on 28 July 2022) containing yeast, fish oils, *Chlorella* and various HUFAs reproducing the composition of natural marine snow in a suspension of particles sizing 5–10 μm [15]. Larvae were filtered every two days onto a 60 μm mesh sieve and transferred to a renewed culture medium using a Pasteur pipette. At the same time intervals, two 50 mL samples were collected to evaluate and record the survival rates, check the larval stage progression, the overall health conditions (mobility, malformations) and the gut fullness. During the experiment, seawater samples were collected biweekly and nitrites and phosphates were measured (using a photometer AL 450, Aqualytic, Alkimia Srl), as well as salinity and temperature (see Supplementary Table S1 in the Supplementary Materials).

### 4.2. Statistical Analyses

The survival rates of larvae deriving from two sets of females (pooling of individuals A-B vs. C-D) were calculated using GraphPad Prism 8 software (GraphPad Software, San Diego, CA, USA, www.graphpad.com, accessed 1 September 2021) using the following equation for one phase decay:

(1) Y=(Y0−Plateau)∗exp (−K∗X)+Plateau

where: Y0 is the Y value when X (time) is zero; Plateau is the Y value at infinite times; K is the rate constant, expressed in reciprocal of the X-axis time units. The survival rates obtained with A-B vs. C-D larvae, as well as those obtained in smaller vs. larger tanks, were compared and two-sided *p*-values were obtained by paired *t*-tests.

The slopes obtained from different individuals (A-B vs. C-D) in different types of culture tanks (smaller vs. larger) were compared by linear regression on the data limited to the first week, to avoid any influence of settlement (older larvae settle and the number of swimming individuals is reduced) and to determine the slope of a best-fit line. A value of *p* < 0.05 was chosen as a threshold level for significance. All data have been analyzed with the aid of GraphPad Prism 8.0 software.

### 4.3. RNA Extraction and cDNA Synthesis

About 5000 fertilized eggs from each of the four sea urchin females were collected at the pluteus stage, corresponding to 48 h post-fertilization (hpf). These samples were centrifuged at 3500 relative centrifugal force for 15 min in a swing-out rotor at 4 °C. Embryos were then placed in 60 microliters of the RNA-later (Qiagen, Hilden, Germany) and then frozen in liquid nitrogen. Samples were kept at −80 °C until use. Total RNA was extracted using *Aurum Total RNA Mini Kit* (Bio–Rad, Hercules, CA, USA), according to the manufacturer’s instructions. The amount of total RNA extracted was estimated by the absorbance at 260 nm and the purity by 260/280 and 260/230 nm ratios, using a NanoDrop spectrophotometer (ND–1000 UV–VIS Spectrophotometer; NanoDrop Technologies, Wilmington, DE, USA). The integrity of RNA was evaluated by observing the rRNA subunits (28S and 18S) on agarose gel electrophoresis. For each sample, 1000 ng of total RNA was retrotranscribed with an *iScript cDNA Synthesis kit* (Bio–Rad, Milan, Italy), following the manufacturer’s instructions.

### 4.4. Real Time qPCR Experiments

The variations in the expression of twenty-three genes were followed by *Real Time qPCR* (see Supplementary Table S2 in the Supplementary Materials for their biological functions and Supplementary Table S3 for the sequence of the primers) in sea urchin embryos deriving from each of the four females collected at the pluteus stage, reached at 48 hpf. cDNA (1 μL) was used as a template in a reaction containing a final concentration of 0.3 mM for each primer and 1× FastStart SYBR Green master mix (total volume of 10 μL) (Applied Biosystems, Monza, Italy). PCR amplifications were performed in a ViiATM7 *Real Time PCR* System (Applied Biosystems, Monza, Italy) thermal cycler using the following thermal profile: 95 °C for 10 min, one cycle for cDNA denaturation; 95 °C for 15 s and 60 °C for 1 min, 40 cycles for amplification; one cycle for final elongation at 72 °C for 5 min; one cycle for melting curve analysis (from 60 °C to 95 °C) to verify the presence of a single product. Each assay included a no-template control for each primer pair. To capture intra-assay variability, all *Real-Time qPCR* reactions were carried out in triplicate. Fluorescence was measured using ViiATM7 software (Applied Biosystems, Monza, Italy). For all *Real-Time qPCR* experiments the data of each cDNA sample were normalized with the mRNA level of the ubiquitin [5] and 18S rRNA [64] genes, used as housekeeping genes. The expression of each gene was analyzed and normalized against the internal control by REST program (Relative Expression Software Tool), based on the Pfaffl method, reporting the expression values of the genes of interest with respect to the housekeeping genes, using each time one of four different females as the reference group [65,66]. Relative expression ratios above two cycles were considered significant. Experiments are means between duplicates. Statistical analysis was performed using GraphPad Prism version 8.00 for Windows (GraphPad Software, San Diego, CA, USA).

The network analysis on genes under analysis was performed by STRING [67], which aims to identify relationships on the basis of associated functions and data mining from experimental studies reported in the literature. Since sea urchin genes are not annotated in the STRING database, human orthologues were used to search for *P. lividus* genes.

## Figures and Tables

**Table 1:** Paired *t*-test (two-tailed *p* value) to compare the larval survivorships (A-B vs. C-D) in smaller and larger tanks. ** indicates *p* < 0.01.

Paired *t* Test	Smaller	Larger
***p*** **value**	0.0830	0.1169
***p*** **value summary**	n.s.	n.s.
**t**	1.980	1.758
**df**	8	8
Mean of differences (B-A)	−21.93	−8.778
SD of differences	33.22	14.98
SEM of differences	11.07	4.994
95% confidence interval	−47.46 to 3.610	−20.29 to 2.738
R squared (partial eta squared)	0.3289	0.2786
Correlation coefficient (r)	0.7608	0.8595
*p* value (one tailed)	0.0086	0.0015
*p* value summary	**	**
Significantly effective pairing	Yes	Yes

## Data Availability

Not applicable.

## Supplementary Materials

The following supporting information can be downloaded at: https://www.mdpi.com/article/10.3390/ijms232112790/s1.
