# Supplementary material for: Gene Expression Detects the Factors Influencing the Reproductive Success and the Survival Rates of Paracentrotus lividus Offspring
Source: Int J Mol Sci. 2022 Oct 24;23(21):12790. doi: 10.3390/ijms232112790 (PMC9659156; doi:10.3390/ijms232112790)
Supplement: Supplementary file 1 [file ijms-23-12790-s001.zip › ijms-1866534-supplementary.pdf]

Supplementary Material

Table S1. Abiotic descriptors of water quality for "small" and "large" tank sizes. The trends of these descriptors were recorded in small-size tanks to keep the main organic pollutants under control and keep them lower than the values detectable by the employed instruments, besides a single time, when PO_4 reached the maximum value, corresponding to 0.4 mg/L. Concerning the large size experimental tanks, the values were consistently kept lower than the detectable limit. Only at one time PO_4 and NO_2 reached slightly higher values (0.02 mg/L and 0.14 mg/L respectively).

	Measured abiotic descriptor	Max value (mg/L)	Min value (mg/L)
Small size			
	NO_2	<0.01	< 0.01
	PO_4	0.4 (24/02/2021)	<0.05
	Temperature	18°C	17°C
Large size			
	NO_2	0.02	< 0.01
	PO_4	0.14 (22/02/2021)	<0.05
	Temperature	18°C	17°C

Table S2. Biological functions of the examined genes.

Name of the gene	Function
ARF1	*ADP-ribosylation factor 1;* GTP-binding protein that functions as an allosteric activator of the cholera toxin catalytic subunit, an *ADP- ribosyltransferase.* Involved in protein trafficking among different compartments. Modulates vesicle budding and uncoating within the Golgi complex. Deactivation induces the redistribution of the entire Golgi complex to the endoplasmic reticulum, suggesting a crucial role in protein trafficking. In its GTP-bound form, its triggers the association with coat proteins with the Golgi membrane. The hydrolysis of ARF1-bound GTP, which is mediated by ARFGAPs [...] (181aa)
CASP8	*Caspase-8;* most upstream protease of the activation cascade of caspases responsible for the TNFRSF6/FAS mediated and TNFRSF1A induced cell death. Binding to the adapter molecule FADD recruits it to either receptor. The resulting aggregate called death-inducing signaling complex (DISC) performs *CASP8* proteolytic activation. The active dimeric enzyme is then liberated from the DISC and free to activate downstream apoptotic proteases. Proteolytic fragments of the N-terminal propeptide (termed CAP3, CAP5 and CAP6) are likely retained in the DISC. Cleaved and activates CASP3, CASP4, CASP6, […] (538aa)
CASP7	*Caspase-7;* Involved in the activation cascade of caspases responsible for apoptosis execution. Cleaves and activates sterol regulatory element binding proteins (SREBPs). Proteolytically cleaves poly(ADP-ribose) polymerase (PARP) at a '216-Asp-|-Gly- 217' bond. Overexpression promotes programmed cell death (388aa)
MT-CYB	*Cytochrome b;* Component of the ubiquinol-cytochrome c reductase complex (complex III or cytochrome b-c1 complex) that is part of the mitochondrial respiratory chain. The b-c1 complex mediates electron transfer from *ubiquinol* to *cytochrome c*. Contributes to the generation of a proton gradient across the mitochondrial membrane that is then used for ATP synthesis (380aa)
DNMT1	*DNA (cytosine-5)-methyltransferase 1;* Methylates CpG residues. Preferentially methylates hemimethylated DNA. Associates with DNA replication sites in S phase maintaining the methylation pattern in the newly synthesized strand, that is essential for epigenetic inheritance. Associates with chromatin during G2 and M phases to maintain DNA methylation independently of replication. It is responsible for maintaining methylation patterns established in development. DNA methylation is coordinated with methylation of histones. Mediates transcriptional repression by direct binding to *HDAC2*. In a [...] (1632aa)

ERCC3	TFIIH basal transcription factor complex helicase XPB subunit; ATP-dependent 3'-5' DNA helicase, component of the core- TFIIH basal transcription factor, involved in nucleotide excision repair (NER) of DNA and, when complexed to CAK, in RNA transcription by RNA polymerase II. Acts by opening DNA either around the RNA transcription start site or the DNA damage (782aa)
GLUL	*Glutamine synthetase;* This enzyme has 2 functions: it catalyzes the production of glutamine and 4-aminobutanoate (gamma-aminobutyric acid, GABA), the latter in a pyridoxal phosphate-independent manner (By similarity). Essential for proliferation of fetal skin fibroblasts; Belongs to the glutamine synthetase family (373aa)
GRHPR	*Glyoxylate reductase/hydroxypyruvate reductase;* Enzyme with *hydroxy-pyruvate reductase, glyoxylate reductase* and *D-glycerate dehydrogenase* enzymatic activities. Reduces hydroxypyruvate to D-glycerate, glyoxylate to glycolate oxidizes D-glycerate to hydroxypyruvate (328aa)
FKBP4	*Peptidyl-prolyl cis-trans isomerase FKBP4;* Immunophilin protein with PPIase and co-chaperone activities. Component of steroid receptors heterocomplexes through interaction with *heat-shock protein 90 (HSP90).* May play a role in the intracellular trafficking of heterooligomeric forms of steroid hormone receptors between cytoplasm and nuclear compartments. The isomerase activity controls neuronal growth cones via regulation of *TRPC1* channel opening. Acts also as a regulator of microtubule dynamics by inhibiting *MAPT/TAU* ability to promote microtubule assembly. May have a protective role a [...] (453aa)
HSPD1	*60 kDa heat shock protein,* mitochondrial; Chaperonin implicated in mitochondrial protein import and macromolecular assembly. Together with *Hsp10*, facilitates the correct folding of imported proteins. May also prevent misfolding and promote the refolding and proper assembly of unfolded polypeptides generated under stress conditions in the mitochondrial matrix. The functional units of these chaperonins consist of heptameric rings of the large subunit *Hsp60*, which function as a back-to-back double ring. In a cyclic reaction, *Hsp60* ring complexes bind one unfolded substrate protein per rin [...] (573aa)
HSPA4	Heat shock protein family A member 4; Belongs to the heat shock protein 70 family (840aa)
HIF1A	*Hypoxia-inducible factor 1-alpha*; Functions as a master transcriptional regulator of the adaptive response to hypoxia. Under hypoxic conditions, activates the transcription of over 40 genes, including erythropoietin, glucose transporters, glycolytic enzymes, vascular endothelial growth factor, HILPDA, and other genes whose protein products increase oxygen delivery or facilitate metabolic adaptation to hypoxia. Plays an essential role in embryonic vascularization, tumor angiogenesis and pathophysiology of ischemic disease. Binds to core DNA sequence 5'-[AG]CGTG-3' within the hypoxia res [...] (850aa)

RELA	*Transcription factor p65;* NF-kappa-B is a pleiotropic transcription factor present in almost all cell types and is the endpoint of a series of signal transduction events that are initiated by a vast array of stimuli related to many biological processes such as inflammation, immunity, differentiation, cell growth, tumorigenesis and apoptosis. NF-kappa-B is a homo- or heterodimeric complex formed by the Rel-like domain-containing proteins *RELA/p65, RELB, NFKB1/p105, NFKB1/p50, REL* and *NFKB2/p52* and the heterodimeric p65-p50 complex appears to be most abundant one. The dimers bind at kapp [...] (551aa)
PARP1	*60 kDa heat shock protein, mitochondrial;* Chaperonin implicated in mitochondrial protein import and macromolecular assembly. Together with *Hsp10,* facilitates the correct folding of imported proteins. May also prevent misfolding and promote the refolding and proper assembly of unfolded polypeptides generated under stress conditions in the mitochondrial matrix. The functional units of these chaperonins consist of heptameric rings of the large subunit *Hsp60,* which function as a back-to-back double ring. In a cyclic reaction, *Hsp60* ring complexes bind one unfolded substrate protein per rin [...] (573aa)
MAPK14	*Mitogen-activated protein kinase 14;* Serine/threonine kinase which acts as an essential component of the *MAP kinase* signal transduction pathway. *MAPK14* is one of the four *p38 MAPKs* which play an important role in the cascades of cellular responses evoked by extracellular stimuli such as proinflammatory cytokines or physical stress leading to direct activation of transcription factors. Accordingly, *p38 MAPKs* phosphorylate a broad range of proteins and it has been estimated that they may have approximately 200 to 300 substrates each. Some of the targets are downstream kinases which are a [...] (360aa)
SDHB	*Succinate dehydrogenase [ubiquinone]* iron-sulfur subunit, mitochondrial; Iron-sulfur protein (IP) subunit of *succinate dehydrogenase (SDH*) that is involved in complex II of the mitochondrial electron transport chain and is responsible for transferring electrons from succinate to ubiquinone (*coenzyme Q*) (280aa)
TP53	*Cellular tumor antigen p53*; Acts as a tumor suppressor in many tumor types; induces growth arrest or apoptosis depending on the physiological circumstances and cell type. Involved in cell cycle regulation as a trans-activator that acts to negatively regulate cell division by controlling a set of genes required for this process. One of the activated genes is an inhibitor of cyclin-dependent kinases. Apoptosis induction seems to be mediated either by stimulation of BAX and FAS antigen expression, or by repression of Bcl-2 expression. In cooperation with mitochondrial PPIF is involved in [...] (393aa)
YWHAE	*14-3-3 protein epsilon;* Adapter protein implicated in the regulation of a large spectrum of both general and specialized signaling pathways. Binds to a large number of partners, usually by recognition of a phosphoserine or phosphothreonine motif. Binding generally results in the

	modulation of the activity of the binding partner (By similarity). Positively regulates phosphorylated protein HSF1 nuclear export to the cytoplasm; Belongs to the 14-3-3 family
BMP7	*Bone morphogenetic protein 7;* Induces cartilage and bone formation. May be the osteoinductive factor responsible for the phenomenon of epithelial osteogenesis. Plays a role in calcium regulation and bone homeostasis; Bone morphogenetic proteins
Nectin-1	*PVRL1 - Nectin-1;* Promotes cell-cell contacts by forming homophilic or heterophilic trans-dimers. Heterophilic interactions have been detected between *NECTIN1* and *NECTIN3* and between *NECTIN1* and *NECTIN4*. Has some neurite outgrowth-promoting activity; Belongs to the nectin family [a.k.a. *HVEC, NECTIN1, PRR1, nectin-1, Nectin-1,* [...]
SM30	30 kDa spicule matrix protein; Matrix protein of the sea urchin embryo spicule. The function of the matrix proteins is to direct crystal growth in certain orientations and inhibit growth in others [a.k.a. SM30, NP_999766.1, SPU_000827]
SM50	50 kDa spicule matrix protein; Major matrix protein of the sea urchin embryo spicule. The function of the matrix proteins is to direct crystal growth in certain orientations and inhibit growth in others; Belongs to the SM50 family
uni	Adhesion protein that plays a role in the organization of adherens. The protein is a calcium-independent cell-cell adhesion molecule that belongs to the immunoglobulin superfamily and has 3 extracellular immunoglobulin-like loops, a single transmembrane domain (in some isoforms), and a cytoplasmic region

Table S3. Sequence information on the primers used for Real Time qPCR.

Gene name		Acronym	Primer (5'→3')	Sequences (5'→3')	References
Stress	***ADP-ribosylation factor 1***	*ARF1*	ADP_F2	CTGGTGCATCAGTAAACTAC	Esposito et al., 2020
			ADP_R2	CGTCGTAAGAGCAGGAACG	
	caspase-8	*CASP8*	Cas8_Pl_F2	GATACGACGAGCAGCGCAACATCTAG	Romano et al., 2011
			Cas8_Pl_R2	CTAGCATCATCCACTCTCATCCACTGCAC	
	Sp-Cspe3/7L	*caspase 3/7*	Sp_Cas7_F2L	CTTCTTTATACAGGCATGCAGAGGCACCCAGATG	Ruocco et al., 2016
			Sp_Cas7_F2L	CCGCCACGAGTAGTAACCTGGCGTTGATGAAAATGC	
	Cytochrome b	*cytb*	Pl_Cyt_F1	GGGATACGTATTAGTCTGAGG	Marrone et al., 2012
			Pl_Cyt_R1	CGAGTTAGGGTGGCATTGTC	
	DNA-methyltransferase 1	*MTase*	Pl_Met1_F1	GATCTCGTCAGACGATAGAAG	Marrone et al., 2012
			Pl_Met1_R1	CTCTTGCTGTGTTAGCATTG	
	ERCC excision repair 3	*ERCC3*	Pl_XPB_ERCC3_F	CAGGTTTCATCCCATGGTGGATCA	Ruocco et al., 2019a
			Pl_XPB_ERCC3_F	ATACTCCTCCGCTGCAGCACCT	
	Glutamine synthetase	*GS*	Pl_GS_F1	GTGTCGGACCGATATCTGAC	Marrone et al., 2012
			Pl_GS_R1	CTCCGATTGATCCGTACTCG	
	Glyoxylate reductase/hydroxypyruvate reductase	*GRHPR*	GLR_F1	GGCTCACAACAGATGAAGTAG	Esposito et al., 2020
			GLR_R1	CTTGGCGTATCTTCGTTCTC	
	Heat Shock Protein 56	*hsp56*	Pl_hsp56_F1	GGAGCTATGCTAAGGACATC	Marrone et al., 2012
			Pl_hsp56_R1	CTACAGCCTTAGCGACAGTG	
	Heat Shock Protein 60	*hsp60*	Pl_hsp60_F1	GAATATCCAGTGTACTCCGAC	Marrone et al., 2012
			Pl_hsp60_R1	GCATCAGCTAAGAGGTCAAC	
	Heat Shock Protein 70	*hsp70*	Pl_hsp70_Up	CAGAACCACGCCCAGCTATG	Marrone et al., 2012
			Pl_hsp70_Rev	GCTTGGATGCTACTATCGTTG	

	Hypoxia inducible factor 1-alpha	*HIF1A*	Sp_HIF1A_F1	CGATAGAAGAGATCATCGACTC	Varrella et al., 2016a
			Sp_HIF1A_R1	GTAGTCGTAGATGCTCTGGC	
	Nuclear factor kappalight-chain-enhancer of activated B cells	*NF-kB*	Pl-NF-kB_F	TCCCATGGAGGACTGCCGTGTCA	Russo et al., 2014
			Pl-NF-kB_R	TCGTTGGTTACCAAGGAGACCACA	
	Poly(ADP-ribose) polymerase 1	*PARP-1*	pADP_F1	GGCAATGATGCTGTCTGTAG	Esposito et al., 2020
			pADP_R1	CAGTTACAGCACTGGTTTCATC	
	p38 mitogen-activated protein kinase	*p38 MAPK*	Pl_p38_F1	GTGATCAGCTTGCTTGACTG	Marrone et al., 2012
			Pl_p38_R1	GTAGATGAGGAACTGGACGTG	
	Succinate dehydrogenase	*SDH*	SDH_F2	GGTGAGTAGGAAATTCACAGTG	Esposito et al., 2020
			SDH_R2	CTCACGTCGGAATGTCTTCG	
	Tumor protein p53	*p53*	Sp_p53_F1	GCGTTGGTGGATCATACTGG	Varrella et al., 2016a
			Sp_p53_R1	GATCTTGGTCTGAGCGTAGTG	
	14-3-3 epsilon protein	*14-3-3 ε*	Pl_Eps_F1	CGGATAGATACAATGACATGG	Marrone et al., 2012
			Pl_Eps_R1	GCTGACTGTATGCAATGCTG	
Skeletogenesis	***Bone morphogenetic protein 5-7***	*BMP5-7*	Pl_BMP_F1	TGGCAGGAATGGATCATCGC	Marrone et al., 2012
			Pl_BMP_F1	GAGTGTCTGCACGATGGCGTG	
	Nectin	*Nec*	Pl_Nec_F1	CAAGCACAGCTGGGAATGG	Marrone et al., 2012
			Pl_Nec_R1	GGTCATTTGTTCTTGCACTC	
	Spicule matrix protein 30	*SM30*	Pl_SM30_F1	TTGGGTTCAGTTGGAGAACC	Marrone et al., 2012
			Pl_SM30_R1	GTTTCGTTGTCTTCGGGGTA	
	Spicule matrix protein 50	*SM50*	Pl_Sm50_F1	GATGGCACACCAGCTTATCC	Marrone et al., 2012
			Pl_Sm50_R1	CTGACGCTTCATGACTGGAG	
	Univin	*uni*	Pl_Uni_F1	ACTGGATCATCGCTCCGATG	Marrone et al., 2012
			Pl_Uni_R1	CATCGGCATCCACAAGCTTC	

	Metallothionein 8	*MT8*	MT8 For	GATGGTTGTCGTCGCTCCTAACA	Ragusa et al., 2013
			MT8 Rev	TCAAGAAAGGCTGGTATCAAATCTGAC	
Reference genes	***Ubiquitin***	*Ubi*	Ubi_Pl_For1	CACAGGCAAGACCATCACACTCGAGGTC	Romano et al., 2011
			Ubi_Pl_Rev1	GAGAGAGTGCGACCATCCTCGAGTTGC	
	18S ribosomal RNA	*18S rRNA*	18S rRNA For	GAATGTCTGCCCTATCAACTTTCG	Ragusa et al., 2013
			18S rRNA Rev	TTGGATGTGGTAGCCGTTTCTC	

Table S4. Fold change values of twenty-three genes involved in stress responses and skeletogenesis analysed by *Real-Time qPCR* in embryos at the pluteus stage deriving from A, B, C and D females, respect to the control. Up-regulated and down-regulated genes were highlighted in red and blue, respectively.

	Gene	A	B	C	D
Stress	*ARF1*	0.4	-0.4	-3.5	1.3
	CASP8	-0.9	-2.1	-5.5	-2.7
	caspase 3/7	0.2	-1.2	-3.5	-0.5
	cytb	-0.3	1.3	2.0	1.3
	Mtase	-1.2	1.2	0.2	0.3
	ERCC3	0.8	-1.4	-3.9	-3.2
	GS	-0.7	0.8	-1.7	-1.4
	GRHPR	-1.1	1.1	-2.4	0.7
	hsp56	0.0	-1.0	-0.2	0.9
	hsp60	0.9	-0.6	-2.4	-2.8
	hsp70	0.1	0.2	-6.4	-2.9

	HIF1A	0.3	-0.7	-1.9	-0.6
	NF-kB	0.0	1.0	0.0	0.0
	PARP-1	0.8	-1.0	-3.0	-1.0
	p38MAPK	-0.3	0.6	-1.7	-2.3
	SDH	0.2	-0.4	-1.4	-2.7
	p53	0.4	-0.3	-4.8	-4.4
	14-3-3 ε	0.1	0.1	-5.5	-3.4
Skeletogenesis	*BMP5-7*	1.2	1.6	-2.2	-1.1
	Nec	-0.8	-0.9	-0.0	0.2
	SM30	0.9	-1.0	-3.9	-1.0
	SM50	-0.1	-0.2	-4.0	-7.3
	uni	0.2	0.0	3.4	4.0
